# Extrusion Printing of Surface‐Functionalized Metal‐Organic Framework Inks for a High‐Performance Wearable Volatile Organic Compound Sensor

**DOI:** 10.1002/advs.202400207

**Published:** 2024-04-24

**Authors:** Xiao Wang, Hao Qi, Yuzhou Shao, Mingming Zhao, Huayun Chen, Yun Chen, Yibin Ying, Yixian Wang

**Affiliations:** ^1^ School of Biosystems Engineering and Food Science Zhejiang University Hangzhou 310058 P. R. China; ^2^ Key Laboratory of Intelligent Equipment and Robotics for Agriculture of Zhejiang Province Hangzhou 310058 P. R. China; ^3^ State Key Laboratory of Rice Biology Zhejiang University Hangzhou 310058 P. R. China; ^4^ Key Laboratory of Molecular Biology of Crop Pathogens and Insects, Institute of Biotechnology Zhejiang University Hangzhou 310058 P. R. China; ^5^ ZJU‐Hangzhou Global Scientific and Technological Innovation Center Hangzhou 310058 P. R. China

**Keywords:** extrusion printing, metal‐organic framework, surface functionalization, volatile organic compounds, wearable colorimetric sensor

## Abstract

Wearable sensors hold immense potential for real‐time and non‐destructive sensing of volatile organic compounds (VOCs), requiring both efficient sensing performance and robust mechanical properties. However, conventional colorimetric sensor arrays, acting as artificial olfactory systems for highly selective VOC profiling, often fail to meet these requirements simultaneously. Here, a high‐performance wearable sensor array for VOC visual detection is proposed by extrusion printing of hybrid inks containing surface‐functionalized sensing materials. Surface‐modified hydrophobic polydimethylsiloxane (PDMS) improves the humidity resistance and VOC sensitivity of PDMS‐coated dye/metal‐organic frameworks (MOFs) composites. It also enhances their dispersion within liquid PDMS matrix, thereby promoting the hybrid liquid as high‐quality extrusion‐printing inks. The inks enable direct and precise printing on diverse substrates, forming a uniform and high particle‐loading (70 wt%) film. The printed film on a flexible PDMS substrate demonstrates satisfactory flexibility and stretchability while retaining excellent sensing performance from dye/MOFs@PDMS particles. Further, the printed sensor array exhibits enhanced sensitivity to sub‐ppm VOC levels, remarkable resistance to high relative humidity (RH) of 90%, and the differentiation ability for eight distinct VOCs. Finally, the wearable sensor proves practical by in situ monitoring of wheat scab‐related VOC biomarkers. This study presents a versatile strategy for designing effective wearable gas sensors with widespread applications.

## Graphical abstract

A wearable VOC sensor array is developed using extrusion printing of surface‐functionalized sensing materials‐based inks. Surface‐modified PDMS enhances humidity resistance and VOC sensitivity, and improves the dispersibility in PDMS liquid matrix for high‐quality inks. The extrusion‐printed sensor array exhibits satisfactory mechanical stability and efficient VOC‐profiling ability. The wearable sensor demonstrates its practicality in non‐invasive detection of wheat scab.

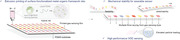

## 1 Introduction

Selective and accurate detection of VOCs has garnered substantial attention as a potent way for disease diagnosis,^[^ ^1^, ^2^, ^3^, ^4^ ^]^ environmental monitoring,^[^ ^5^, ^6^, ^7^ ^]^ and food quality assurance.^[^ ^8^, ^9^, ^10^ ^]^ Especially, VOCs are well‐known as crucial biomarkers for the early diagnosis of plant diseases.^[^ ^11^, ^12^ ^]^ Unlike typical laboratory VOC assays, such as gas chromatography‐mass spectrometry (GC‐MS), wearable VOC sensors offer an effective alternative for easy‐operation, non‐invasive, and real‐time monitoring of VOC markers.^[^ ^13^, ^14^, ^15^, ^16^, ^17^ ^]^ Given the complex mixture of the emitted VOCs, it is imperative to use array‐based sensors that emulate the mammalian olfactory system with desirable specificity for selective VOC profiling.^[^ ^18^, ^19^, ^20^ ^]^ As one of the cross‐reactive sensors, the colorimetric sensor array has been widely employed as a method of visual VOC detection due to its rapid response, costeffectiveness, and portability.^[^ ^21^, ^22^ ^]^ Overall, there is a growing need and interest in developing wearable colorimetric VOC sensors that enable in situ VOC sensing and real‐time monitoring of physiological conditions.^[^ ^14^, ^16^ ^]^ However, there is limited existing research. Indeed, several challenges need to be addressed when it comes to developing wearable sensors for real‐world applications. These challenges encompass the need for exceptional sensitivity to detect relatively low‐concentration VOC emissions,^[^ ^23^ ^]^ favorable resistance to environmental interferences,^[^ ^4^, ^24^ ^]^ and mechanical robustness to ensure compatibility with various interfaces.^[^ ^25^, ^26^, ^27^ ^]^

The principle underlying colorimetric sensors is based on chemically reactive dyes that undergo a color change when exposed to specific VOCs, thus enabling visual detection of VOCs.^[^ ^21^, ^22^ ^]^ To enhance the sensing performance of traditional colorimetric sensors, a promising approach has emerged, involving the integration of nanomaterials with gas‐sensitive dyes. In this approach, dyes are typically encapsulated within porous nanostructures, such as MOFs,^[^ ^28^, ^29^ ^]^ sol‐gel silica,^[^ ^30^, ^31^, ^32^ ^]^ and graphene oxide (GO).^[^ ^32^ ^]^ This incorporation significantly increases the surface area, thereby improving the efficiency of VOC adsorption and further enhancing the sensitivity of VOC detection.^[^ ^33^, ^34^, ^35^ ^]^ Additionally, the surface modification of sensing sites has proven to be an effective strategy.^[^ ^4^, ^36^, ^37^, ^38^ ^]^ By introducing hydrophobic materials onto the sensor surface, such as PDMS^[^ ^36^, ^37^, ^38^ ^]^ and covalent organic frameworks (COFs),^[^ ^4^ ^]^ the resistance to interferences is greatly improved. The hydrophobic modification facilitates the identification of VOC targets while reducing reactions with other interfering substances, particularly water molecules in the surrounding air.^[^ ^4^, ^36^, ^37^, ^38^ ^]^ In conclusion, the utilization of porous nanomaterials and the development of surface modification offer an effective way to develop novel gas‐sensing materials for elevating the sensing performance in VOC detection.

However, the powdered gas‐sensing materials, which are inherently fragile, suffer from poor processability, severely limiting their application in the field of flexible and stretchable sensors. Inspired by the mixed‐matrix membrane technology,^[^ ^39^, ^40^, ^41^ ^]^ efforts have been made to combine powdered sensing materials with flexible polymers to develop practical sensors.^[^ ^39^, ^42^, ^43^, ^44^, ^45^, ^46^, ^47^, ^48^ ^]^ While the addition of polymers improves processability, excessive polymers tend to obstruct sensing sites when the amount of sensing particles is low, leading to diminished sensing performance.^[^ ^43^, ^44^ ^]^ To address this issue, polymetric surface modification on particles has demonstrated as an effective approach to produce the high particle‐loading film.^[^ ^49^ ^]^ This is due to enhancing the dispersion of particles within a polymer matrix and promoting the interactions between the two parts.^[^ ^50^, ^51^, ^52^ ^]^ Furthermore, owing to their processability, the hybrid liquids consisting of gas‐sensing particles and liquid polymers can be employed as ideal extrusion‐printing inks. Unlike traditional coating methods for sensor preparation,^[^ ^28^, ^53^ ^]^ extrusion printing is an emerging and powerful tool for programmable and precise deposition of inks onto various sensor substrates and further formation of hybrid films.^[^ ^54^, ^55^ ^]^ Therefore, the extrusion printing of hybrid films not only enhances processability but also facilitates the high‐quality production of printed sensors.

Here, we propose a printing strategy to prepare gas‐sensing hybrid film comprising surface‐polymerized sensing materials and flexible polymers, then adopt this strategy to develop a wearable colorimetric sensor for in situ VOC profiling. A typical MOF (UiO‐66(Zr)) was chosen to load dyes and then coated with a hydrophobic PDMS layer for an optimized coating time of 150 minutes. The PDMS coating enhances humidity resistance and heightens VOC sensitivity to the resulting dye/UiO@PDMS (DUP) composites by preventing the ingress of water molecules and capturing VOC molecules. To ensure ease of processing, we combined powdered dye/UiO@PDMS particles with a flexible PDMS polymers to formulate a hybrid PDMS/DUP extrusion‐printing ink. Within this ink, the PDMS coating on particles also plays a crucial role in enhancing particle dispersion in the PDMS matrix, facilitating the creation of films with a high particleloading. Subsequently, the ink was precisely deposited onto a sensor substrate under controlled extrusion‐printing conditions. This process resulted in a thin and uniform film that exhibited excellent substrate adhesion and a high particle loading of 70% by weight. The printed film applied to a flexible PDMS substrate exhibited remarkable flexibility and stretchability without compromising sensing performance. Furthermore, the printed nine‐element sensor array demonstrated enhanced sensitivity for sub‐ppm VOC detection, remarkable resistance to humidity (up to 90% RH), and excellent recognition capabilities for eight different types of VOCs. Finally, the wearable sensor arrays were directly attached to the wheat leaves for in situ and stable profiling of the VOC emissions, enabling the accurate and early detection of wheat scab just two days after inoculation.

## 2 Results and Discussion

### 2.1 Polymeric Surface Coating of Dye/MOFs for Humidity‐Independent and Sensitive VOC Sensing

**Figure 1:**
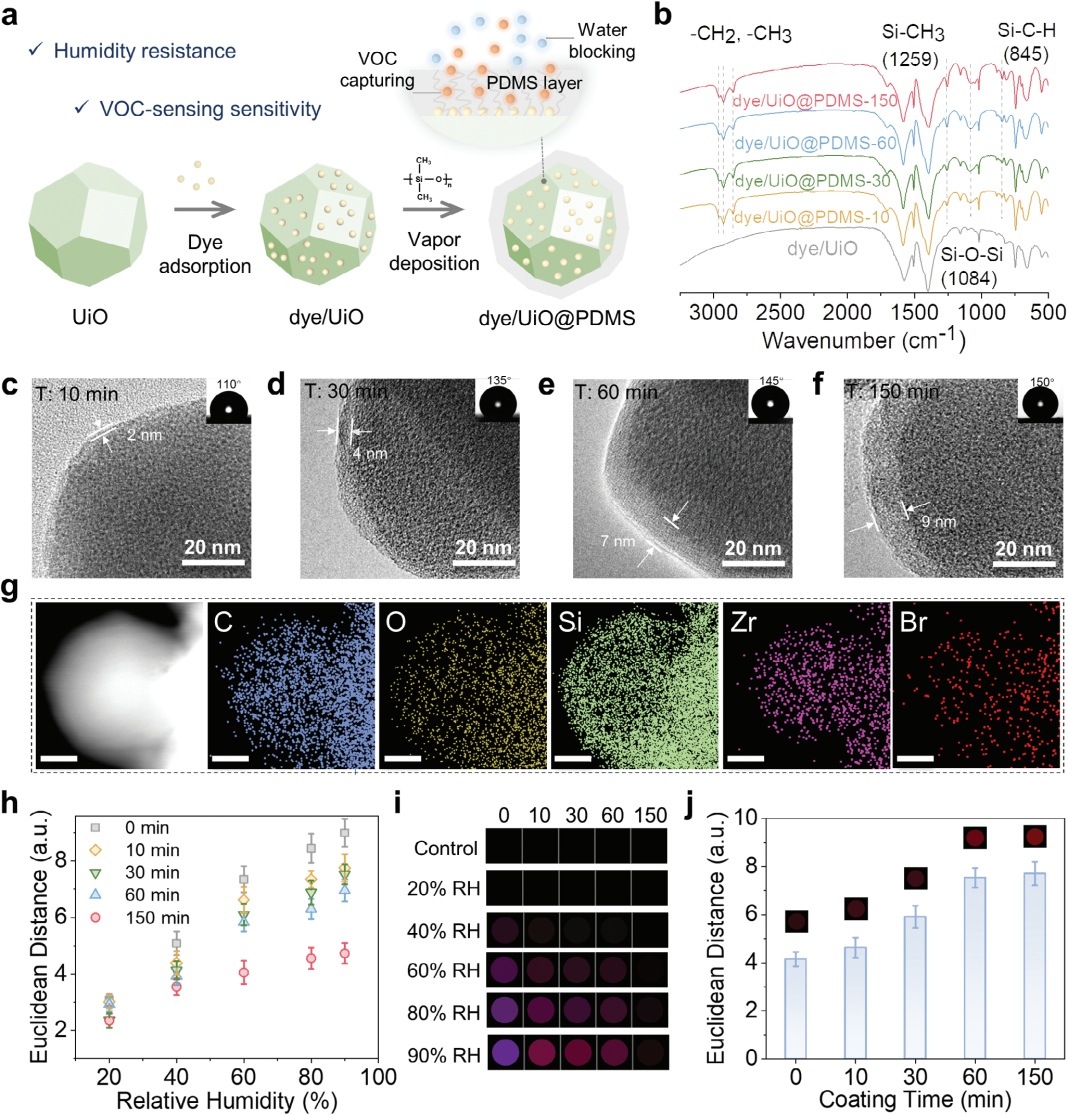
Hydrophobic PDMS coating on dye/MOFs for humidity‐independent and sensitive VOC sensing. a) Illustration of preparing dye/UiO@PDMS core‐shell gas sensing composites and the role of PDMS layer in VOC sensing. b) FTIR spectrums of dye/UiO and dye/UiO@PDMS‐T samples. HRTEM images of dye/UiO@PDMS‐T samples with different coating time of c) 10 min, d) 30 min, e) 60 min, and f) 150 min. Inset: corresponding water contact angle photographs. g) High‐Angle Annular Dark Field Scanning TEM (HAADF‐STEM) image of dye/UiO@PDMS‐150 composites and EDS mapping images of C, O, Si, Zr, and Br. scale bar: 20 nm. The humidity response of dye/UiO@PDMS‐T samples to varying RH, showing in h) plot and i) color differential maps. j) A plot of ED values of dye/UiO@PDMS‐T composites to 10 ppm of 1‐octene‐3‐ol, with corresponding color differential profiles inserted. The dye used in b–j) is bromophenol blue. For display purposes in i) and j), the RGB range was expanded from 3–10 to 0–255. For h) and j), data were presented as means ± SD, with n = 3 independent experiments.

**Figure** **1**a presents a two‐step strategy for preparing the humidity‐resistant and VOC‐sensitive sensing materials. The porous UiO‐66(Zr) (UiO), known for its chemical stability and extensive surface area, was chosen to effectively and uniformly immobilize gas‐sensing dyes by a liquid‐phase adsorption method.^[^ ^56^, ^57^ ^]^ Subsequently, PDMS, a hydrophobic polymer, was coated on the surface of dye/UiO composites by facile chemical vapor deposition (CVD) approach,^[^ ^58^, ^59^, ^60^ ^]^ affording dye/UiO@PDMS sensing composites. In dye/MOFs@PDMS composites, VOC molecules are captured, followed by a chemical reaction with dye molecules, leading to a color change as a sensing signal. Both the permeable PDMS layer and porous MOFs possess the ability to capture VOCs. The hydrophobic and permeable PDMS layer hinders the intrusion of water molecules^[^ ^36^, ^37^, ^38^ ^]^ and facilitates the adsorption of VOC molecules,^[^ ^61^, ^62^, ^63^, ^64^ ^]^ while porous MOFs pre‐concentrate VOC molecules for further enhancing chemical sensing. A synergistic effect between PDMS and MOFs improves the sensitivity and humidity resistance in VOC sensing.

In the CVD process, the thermal degradation of PDMS caused by cleavage of the Si‐O bonds results in the mixture of volatile and lowmolecular‐weight silicone molecules.^[^ ^61^ ^]^ Subsequently, these generated molecules would deposit onto the surface of dye/UiO and undergo further cross‐linking, forming a PDMS layer.^[^ ^58^ ^]^ Therefore, the coating time is a critical parameter in the PDMS coating procedure, determining the condition of PDMS coverage. A series of dye/UiO@PDMS composites were obtained by adjusting the coating time (T) and named dye/UiO@PDMS‐T (T = 10, 30, 60, 150 min). The Fourier transform infrared (FTIR) spectrums of PDMS‐coated composites displayed the stretching bands of the Si‐O‐Si (1084 cm^−1^) assigned to PDMS, confirming the successful formation of PDMS coverage after CVD treatment (Figure 1b). The powder X‐ray diffraction (PXRD) patterns of dye/UiO and dye/UiO@PDMS‐T still retained characteristic peaks corresponding to UiO‐66, indicating that the crystalline nature and structure of UiO‐66 are maintained after the incorporation of dye molecules and the modification of PDMS layer (Figure S1, Supporting Information). Further, the morphological structure of PDMS‐coated samples varying with different coating time were characterized using high‐resolution transmission electron microscopy (HRTEM). With the extension of the coating time, the progressively increasing thickness of the PDMS shell was observed from ∼2 nm to ∼9 nm (Figure 1c–f). Furthermore, water contact angle (CA) measurements were conducted to investigate surface hydrophobization related to the varying thickness of the hydrophobic PDMS shell. The surface became hydrophobic upon the PDMS coating, and the enhancement of PDMS shell thickness resulted in gradually improved hydrophobicity with the CA increasing from 110° to 150° (Figure 1c–f). Moreover, the energy dispersive spectroscopy (EDS) mapping images of dye/UiO@PDMS‐150 showed a larger distribution area of Si element (a characteristic element of PDMS) than that of Zr element (a characteristic element of UiO) and Br element (a characteristic of dye), suggesting the homogeneous distribution of loaded dye molecules and a well‐coated PDMS structure (Figure 1g).

As anticipated, surface modification of dye/UiO by hydrophobic PDMS coating significantly enhances the humidity resistance of gas‐sensing materials. During the VOC sensing process, water molecules are potential targets that compete with VOC molecules to occupy sensing sites and trigger the response. The PDMS coating serves as effective water blocking to protect the active sites of the gas‐sensitive materials for VOC sensing. We performed humidity‐response experiments to evaluate the anti‐humidity effect of dye/UiO@PDMS‐T composites for VOC sensing. As shown in Figure 1h,i, similarly to hydrophilic dye/UiO, dye/UiO@PDMS‐T (T = 10, 30, 60 min) composites showed steadily increasing response to enhancing RH, even the response values (Euclidean distance, ED) are remarkably close. In contrast, dye/UiO@PDMS‐150 exhibited a negligible response to both low RH and high RH. It is worth noting that, although the dye/UiO@PDMS‐T composites are hydrophobic, they still showed different humidity‐resistant performances. That could be explained by the different water adsorption capabilities in reported literatures,^[^ ^64^, ^65^ ^]^ as the water molecules in the environment are vapor phase not liquid phase.^[^ ^36^ ^]^ Furthermore, the dye/UiO@PDMS‐150 composites displayed consistent responses to 10 ppm 1‐octene‐3‐ol at varying RH (ranging from 15% to 90% RH), confirming the capacity to detect VOCs independently of humidity conditions (Figure S2, Supporting Information).

Regarding enhancing sensitivity, the PDMS coating is considered advantageous due to its effective ability to adsorb and enrich VOC molecules. Accordingly, we measured the VOC‐sensing response of dye/UiO@PDMS samples with different coating time (Figure 1j). The increasing response value corresponds to a thicker PDMS shell, which can be attributed to the enhanced VOC capture capability. In addition, the response saturation is reached at the coating time of 60 min, revealing that the proper PDMS shell for VOC capture is achieved. Furthermore, we investigated the influence of the dye loading amount on the sensing performance. The varying dye‐loading amount within the dye/UiO composites was obtained by adjusting the adsorption time, followed by consistent PDMS coating for 150 min. As the adsorption time increased, the dye‐loading amount gradually rose, plateauing at ∼12 h when saturation was reached. The dye/UiO@PDMS composite with a higher dye loading demonstrated a more pronounced response to 10 ppm of 1‐octene‐3‐ol (Figure S3, Supporting Information). Considering both humidity resistance and sensitivity performance, we selected the dye/UiO@PDMS‐150 (dye adsorption time: 12 h; PDMS coating time: 150 min) as the desired sensing material for subsequent experiments.

### 2.2 Extrusion Printing of Hybrid Inks Containing Dye/MOFs@PDMS Composites for Gas‐Sensing Film

**Figure 2:**
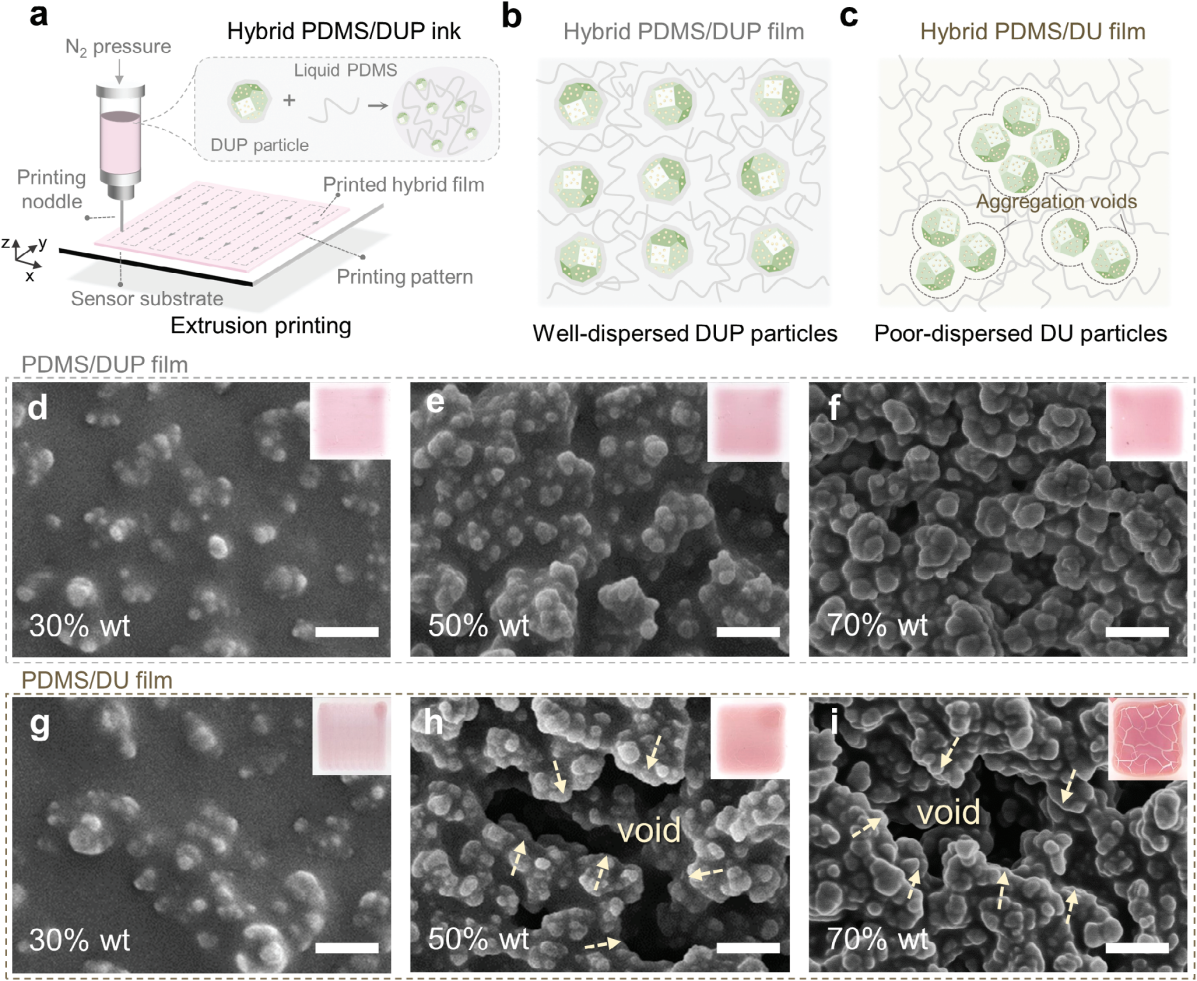
Extrusion printing of hybrid gas‐sensing film containing dye/MOFs@PDMS composites. a) Illustration of the preparation of PDMS/DUP hybrid ink and the process of extrusion printing. Schematic diagram of the distribution of b) dye/UiO@PDMS particles and c) dye/UiO particles in corresponding hybrid film. Top‐view SEM images of d–f) printed PDMS/DUP film and g–i) printed PDMS/DU film with different particle loading. Inset: photos of the corresponding film (3 mm × 3 mm). scale bar: 1 µm. The dye used in d–i) is bromophenol blue.

As illustrated in **Figure** **2**a, we further proposed a printing strategy for preparing hybrid gas‐sensing film, in order to improve the processability of the powdered sensing materials. The first step is to mix the dye/UiO@PDMS‐150 particles with the liquid PDMS matrix to form the hybrid ink, named PDMS/DUP. The prepared PDMS/DUP hybrid ink possessed appropriate viscosity and satisfied rheological properties, suggesting its printability for extrusion printing (Figure S4, Supporting Information). Then, programmable extrusion printing was applied to continuously extrude the appropriate amount of ink, ensuring precise deposition onto the substrate following the predetermined printing path (Figure S5, Supporting Information). The deposited ink can quickly solidify, forming a shaped film attached to the substrate. The printing process can be controlled through adjustments to the printing parameters. Hence, we optimized the printing parameters, including the size of the extrusion noddle and pressure, to obtain a homogeneous film (Figure S6, Supporting Information).

It was worth noting that the surface PDMS coating plays a crucial role in forming homogeneous membranes, particularly for those with high particle loading. In the case of dye/UiO directly dispersed in the PDMS matrix, the poor‐dispersed particles in the polymer matrix tend to aggregate and further trigger the formation of voids in film (Figure 2c). Consequently, the satisfactory film‐forming properties require compromising on particle loading. Unlike pristine dye/UiO particles, the hydrophobic PDMS coating layer endows the dye/UiO@PDMS particles with enhanced dispersity (Figure S7, Supporting Information) and superior affinity to the PDMS matrix. Hence, well‐dispersed PDMS‐coated particles in the PDMS polymer matrix effectively prevent particle aggregation and eliminate interfacial voids (Figure 2b), further promoting the creation of ideal films.

To verify the role of the PDMS coating layer as described above, we prepared two kinds of hybrid inks containing varying particle loading (30 wt%, 50 wt%, and 70 wt%) of dye/UiO (DU) and dye/UiO@PDMS (DUP) particles, and then printed them on PDMS substrate under the same optimized printing conditions. Next, we examined the distinctions between the printed films using optical and scanning electron microscope (SEM) images. When the particle loading is low (30 wt%), there are no discernible distinctions between the two types of films (Figure 2d,g). However, as the particle loading increases (50 wt% and 70 wt%), apparent voids begin to form in the PDMS/DU films (Figure 2h,i). Notably, these voids are responsible for developing cracks in the PDMS/DU (70 wt%) film (Figure 2i). Regardless of the particle loading, the printed PDMS/DUP films maintain uniformity, underscoring the pivotal role of surface‐modified PDMS layer in the film‐forming process (Figure 2d–f).

Due to desired processability of hybrid ink and the controllability of extrusion printing, the printed films with different particle loading are all thin with a thickness of below 10 µm (Figure S8, Supporting Information). The XRD patterns of PDMS/DUP films showed characteristic peaks from dye/UiO@PDMS, demonstrating the crystalline structure still preserves after the film formation (Figure S9, Supporting Information). The water contact angles were also measured, showing the enhancement of hydrophobicity with the increased quantity of hydrophobic particles (Figure S10, Supporting Information). We also adopt this approach to print PDMS/DUP ink on other general sensor substrates, such as polyvinylidene difluoride (PVDF), polyethylene terephthalate (PET), and glass, demonstrating the applicability for diversified printed substrate (Figure S11, Supporting Information). Overall, the proposed approach has been validated as an effective solution to address the challenge of poor processibility issues in solid‐stated sensing materials and enable the fabrication of high particle‐loading film on sensor substrate, thereby showing its potential applicability in printed sensors.

### 2.3 Sensing Capabilities and Mechanical Properties of the Printed Film

The printed film is expected to serve as an exemplary colorimetric gas sensor, offering outstanding sensing capabilities and robust mechanical properties. The favorable sensing properties of incorporated dye/UiO@PDMS composites can be inherited, while the mixed PDMS polymer imparts exceptional flexibility and stretchability to the printed film on a flexible PDMS substrate.

**Figure 3:**
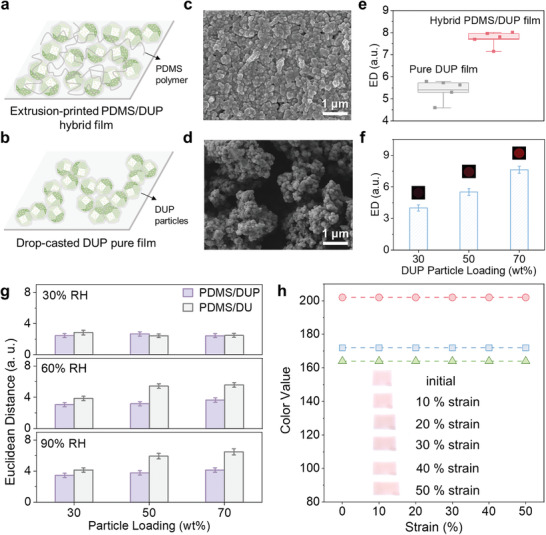
Sensing capabilities and mechanical properties of the printed film. Schematic illustrating the distribution of dye/UiO@PDMS particles in a) printed PDMS/DUP hybrid film and b) drop‐casted DUP pure film. SEM images (top side) of c) PDMS/DUP (70 wt%) hybrid film and d) pure DUP film. The response to 10 ppm of 1‐octene‐3‐ol of e) pure DUP film and printed PDMS/DUP (70 wt%) film, as well as f) printed PDMS/DUP film with different DUP loading, with corresponding color differential profiles inserted. g) The humidity response of varying particle‐loading PDMS/DUP and PDMS/DU films. h) R, G, and B values of the printed PDMS/DUP (70 wt%) film under tensile test from 0 to 50% strain. Inset: optical images of the printed film under strain. The dye used in c–h) is bromophenol blue. For display purposes in f, the RGB range was expanded from 3–10 to 0–255. For e–g), data were presented as means ± SD, with n = 3 independent experiments.

At first, we assessed the VOC‐sensing sensitivity of the printed film (PDMS/DUP (70 wt%)), particularly in comparison with pristine dye/UiO@PDMS particles. Dye/UiO@PDMS particles were deposited on the substrate using conventional drop casting,^[^ ^4^ ^]^ forming pure DUP film. Indeed, this casting approach leads to an uneven distribution of sensing particles with unavoidable aggregation (**Figure** **3**b,d). Consequently, this substantially reduce the available sensing sites for VOC targets, further exerting a negative impact on sensitivity. On the contrary, the integrated polymer within the printed film can address this concern to some extent, facilitating the uniform distribution of sensing particles and guaranteeing more sensing sites for the interaction with VOC molecules (Figure 3a,c). That can be evidenced by the higher VOC‐sensing response of printed film than that of pure DUP film, attributed to increasing sensing sites (Figure 3e). As shown in Figure 3f, the printed film with relatively low particle loading (30% wt) exhibited a lower VOC response than that of pure film, reasonably ascribed to the concealment of the sensing site within PDMS matrix. Notably, this enhanced sensing sensitivity of the printed film is effective under the high‐loading situation. Furthermore, the desired humidity resistance of the printed film made with PDMS/DUP ink was also confirmed by the humidity response experiment, especially when contrasted with the printed films from PDMS/DU ink (Figure 3g). Unlike PDMS/DUP film, PDMS/DU film with high particle loading (50 wt% and 70 wt%) exhibits an obvious enhancing response to increasing RH, indicating its unideal anti‐humidity capability. The observation revealed that the humidity resistance of printed high‐loading film is largely ascribed to the hydrophobic PDMS coating on sensing particles rather than PDMS matrix.

The stretchability of printed film was further verified by tensile tests. As observed in Figure 3h, the printed film (PDMS/DUP (70 wt%)) on flexible PDMS substrate can still maintain structural integrity without crack development when subjected to increased strain ranging from 0% to 50%. The impressive stretchability of printed film can be attributed to the inherent flexibility introduced by incorporating PDMS polymer, as well as the improved adhesion of sensing materials on substrate facilitated by extrusion printing. Correspondingly, the same PDMS/DUP (70 wt%) ink was spin‐coated onto the PDMS substrate at a speed of 400 rpm. In contrast to the printed film, the spin‐coated film exhibited its susceptibility to cracks even at low strains (10%). The poor mechanical behavior is likely attributed to the film’ s high particle loading and relatively greater thickness of 47 µm, surpassing the optimal limit for proper adhesion onto the substrate (Figure S12, Supporting Information). This further highlights the pivotal role of extrusion printing in producing thin and strongly adhesive films onto substrates. More importantly, the printed film consistently maintained stable color values (R, G, B) within 50% tensile strain (Figure 3h), revealing its great potential as a stretchable sensor for reliable colorimetric sensing applications.

### 2.4 Flexible and Stretchable Sensor Array for Detection and Recognition of VOCs

**Figure 4:**
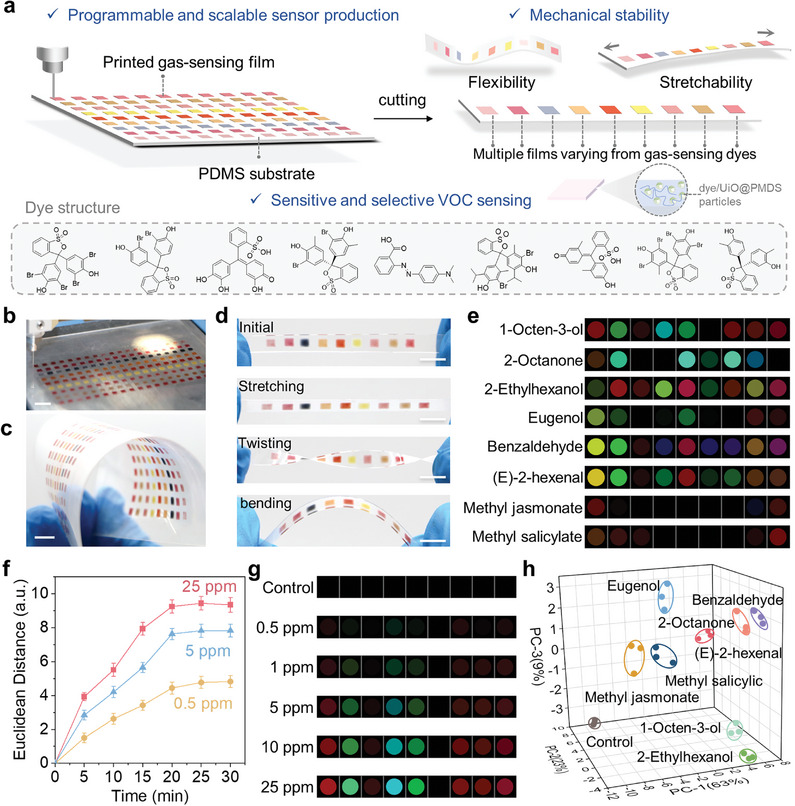
Flexible and stretchable sensor array for visual detection and identification of VOCs. a) Extrusion‐printed flexible and stretchable sensor arrays for selective VOCs profiling. Actual images of b) the extrusion‐printing process for scale preparation of sensors and c) a batch of printed sensor arrays on flexible PDMS substrate. d) Photographs of the initial sensor and the sensor under various mechanical stresses. e) Color differential maps of eight plant VOCs at 10 ppm. f) Response kinetics of the sensor array to 1‐octene‐3‐ol with different concentrations over time. g) Color differential profiles to 1‐octen‐3‐ol under varying concentrations. h) PCA plot of eight plant VOCs and control based on data from three parallel trials, using the first three principal components. For display purposes in e) and g), the RGB range is expanded from 3–10 to 0–255. Scale bar: 10 mm. For f) and h), data were presented as means ± SD, with n = 3 independent experiments.

Inspired by the printed film's remarkable sensing performances and favorable mechanical properties, we adopt the aforementioned approach to prepare a flexible and stretchable sensor array for sensitive detection and selective profiling of VOCs (**Figure** **4**a). The cross‐reaction of the sensor array is the key to highly selective VOC sensing, depending mainly on specific dye‐VOC reactions from multiple gas‐sensing dyes. Nine Brønsted acids/bases dyes with high chemical activity were selected as listed in Table S1 (Supporting Information). Then we prepared the corresponding PDMS/DUP (70 wt%) inks using an identical procedural approach (Figure 4a; Figure S13, Supporting Information). Subsequently, the formulated inks were extrusion‐printed, line by line, onto a flexible PDMS substrate, obtaining a batch of sensor arrays (Figure 4b,c). Hence, this approach allows for the programmable and scalable production of sensor arrays. The sensor array possesses exceptional mechanical properties, enabling it to withstand various mechanical stresses, including stretching, twisting, and bending (Figure 4d; Figure S14, Supporting Information).

To verify the multiplexed sensing capabilities of our sensor arrays, we conducted detection experiments for eight distinct plant VOCs, including four characteristic wheat scab markers (1‐octen‐3‐ol, eugenol, benzaldehyde, and 2‐octanone), two green leaf volatiles (2‐ethyhexol, (E)−2‐hexenal), and two phytohormones (methyl jasmonate and methyl salicylate). These VOCs were identified in prior research and demonstrated to correlate with wheat diseases, thus enabling their potential application in diagnosing wheat disease.^[^ ^4^ ^]^ We first optimized the response time by exposing the sensor arrays to 1‐octen‐3‐ol at various concentrations (0.5, 5, and 25 ppm). The results revealed that the average ED values for nine spots representing sensor response reached saturation after approximately 20 min (Figure 4f). Furthermore, the visible sensor responses to 20‐min VOC exposure were found to be concentration‐dependent, as demonstrated in Figure 4g and Figure S15 (Supporting Information). Figure 4e vividly displayed distinct patterns for each plant VOC at a concentration of 10 ppm, indicating the sensor arrays' ability to differentiate between multiple plant VOCs effectively. To further evaluate the discrimination capability of the sensor arrays, we calculated the limit of recognition (LOR), a critical parameter that assesses the sensors' capacity to identify a specific analyte within a mixture. The LOR of our sensor array was acquired by employing principal component analysis (PCA) for multivariate statistical analysis of the data collected at various concentrations. From the PCA results obtained from the dataset of eight plant VOCs at 10 ppm (Figure 4h), we observed that well‐clustered groups of VOCs could be distinguished from the control group (exposed to N_2_ gas). The response dataset of the eight VOCs and the control group was distinguishable at 1 ppm but exhibited unsatisfied differentiation at 0.5 ppm (Figure S16, Supporting Information). This suggests that the LOR of the sensor arrays for discriminating between the eight VOCs falls within the range of 1 to 0.5 ppm. The wide versatility of our sensor array was further demonstrated by detecting other generic VOCs (Figure S17, Supporting Information). In addition to detecting single VOC, we also identified VOCs mixtures, highlighting the selectivity of our sensor array for mixtures (Figure S18, Supporting Information). Moreover, the excellent humidity resistance of the sensor arrays was demonstrated by the negligible sensor response to water molecules under varying RH (Figure S19, Supporting Information). Furthermore, in comparison to other reported works, the proposed strategy for the flexible sensor array emphasized its advancements in both mechanical properties and sensing capabilities (Table S2, Supporting Information).

### 2.5 In Situ VOC Sensing for Non‐Destructive Detection of Wheat Disease

**Figure 5:**
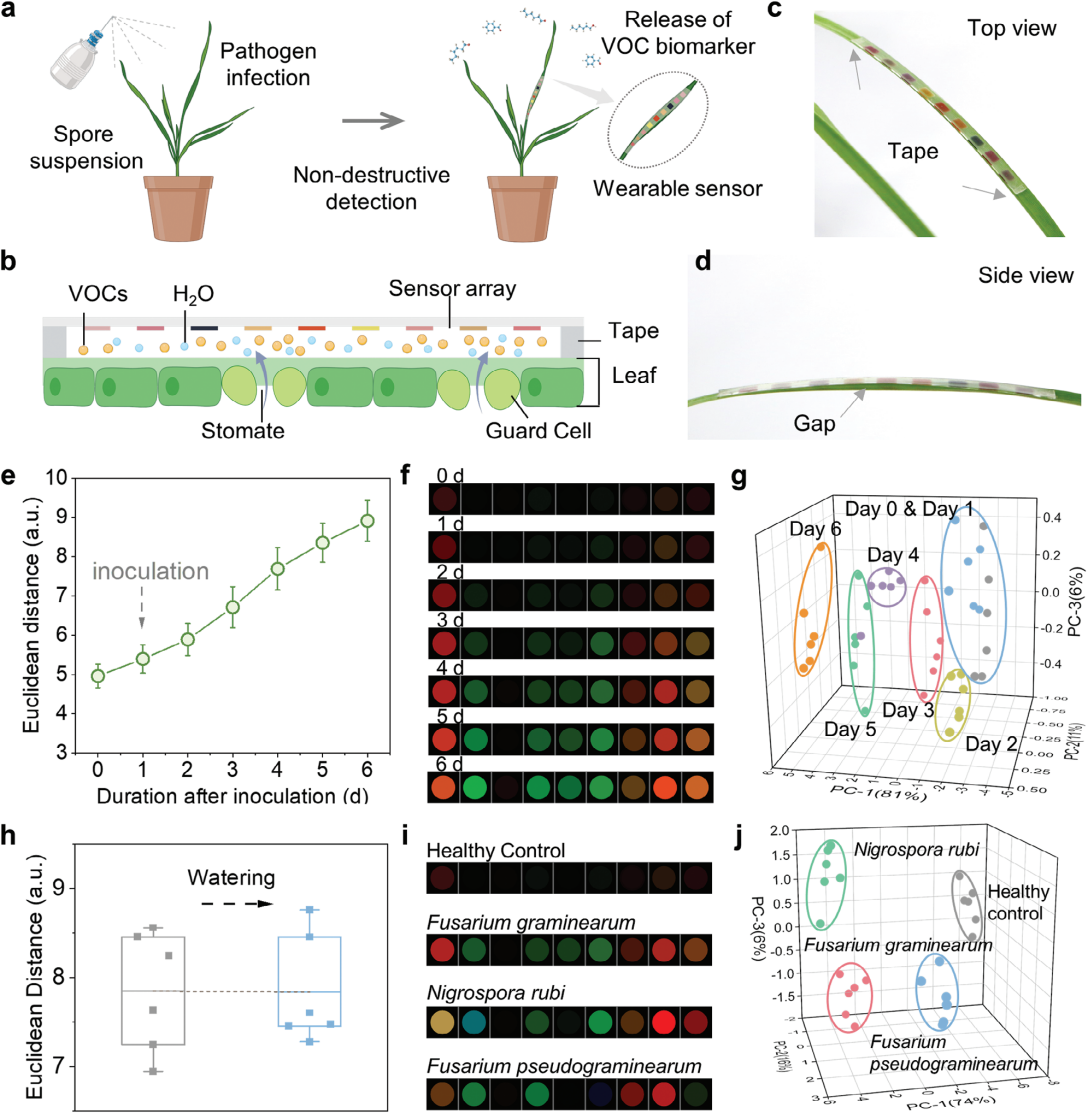
In situ VOC sensing for non‐destructive detection of wheat disease. Schematic illustrating a) the pathogen inoculation for living wheat plants and in situ detection of VOCs released from the infected wheat leaves using the printed sensor array, and b) a side view of the wearable sensor array attached to the wheat leaf. c) Top‐view and d) side‐view photographs of an actual sensor attached to the wheat leaf. e) A plot of average ED value of sensor array, f) Color differential profiles, and g) PCA plot of *F. graminearum* infected wheat samples after inoculation from 0 d to 6 d. h) Average ED values at 4 d before and after watering. i) Color differential profiles and j) PCA plot of healthy control and infected samples by three pathogens on 4 d. n = 6 biologically independent samples for experiments in e–j). For display purposes in f and i, the RGB range is expanded from 3–10 to 0–255. For e) and h), data were presented as means ± SD, with n = 6 independent experiments.

Further, we employed the printed sensor array as a wearable sensor for detecting VOCs released from *Fusarium graminearum* (*F. graminearum*, for wheat scab)‐infected wheat plants. As illustrated in **Figure** **5**a, the wheat plant was infected by *F. graminearum* by spraying the corresponding suspension on the whole wheat plant. Then, the plant status was monitored for the change of VOC emissions by analyzing the colorimetric response of the wearable sensor on wheat leaves.

The lightweight and compact sensor can be easily mounted on the surface of the wheat leaf using double‐sided tape (Figure 5b‐d). The tape with a thickness of ∼0.5 mm maintains a gap between the sensor and the leaf surface, providing a space to diffuse the emitted VOC molecules from leaves (Figure 5c). The in situ sensing producer is shown in Figure 5b. The chemical leaf emissions (including water molecules and VOC molecules) are smoothly released and then rapidly reach the sensing interfaces for colorimetric sensing response. As expected, the sensor array can be applied to the complex environment in practical plant sensing, showing sensitive sensing performance and humidity‐independent for plant status monitoring.

We performed the monitoring experiment for plant VOC emission before and after pathogen infection (0‐6 d). The daily VOC emission was tested by attaching the wearable sensor array to the leaves for 30‐min detection. The responses of the sensor array to *F. graminearum* and the corresponding visible patterns are presented in Figure 5e,f. The average ED values of sensor arrays (Figure 5e) and the VOC profiles (Figure 5f) exhibited a consistent upward trend upon the infection. In contrast to the relatively low response profile of healthy leaves (0 d), the response pattern of infected leaves (1‐6 d) became progressively distinct, reflecting an increasing number of spots and higher intensity (Figure 5f). The heightened response from 0 d to 6 d can be attributed to the increased characteristic emission as the inoculation day increase. The PCA analysis shows that the infected wheat samples at different inoculation stages and healthy samples can be easily distinguished utilizing the first three principal components (Figure 5g). The samples representing 2–6 d were distinctly clustered and separated from those characterizing 1 d and healthy control samples. Furthermore, it is verified that attaching the sensor to the leaf for 30 min does not damage the leaves (Figure S20, Supporting Information). Hence, we deduced that the engineered sensor array is feasible for non‐destructive and early detection of *F. graminearum* within two days after inoculation, before the onset of visible symptoms at 3 d.

The interference resistance of the sensor array was further probed during in situ plant VOC detection. As is known, the release of water vapor from plant leaves could be influenced by various external factors, leading to fluctuations in the humidity within the detection micro‐environment.^[^ ^66^, ^67^ ^]^ Therefore, we respectively detected the plant VOC emissions before and after watering by attaching the sensor to *F. graminearum*‐infected plant leaves. As shown in Figure 5h, within two hours after watering, the sensor response remained consistent with those before, confirming favorable humidity resistance in practical sensing applications.

To assess the specificity of *F. graminearum* detection, we tested two other wheat pathogens, including *Nigrospora rubi* for heat black spots and *Fusarium pseudograminearum* for wheat crown rot. The sensor response of these different pathogen‐infected samples and healthy controls revealed obviously discriminable patterns (Figure 5i), likely attributed to the diverse VOC composition released from leaves. Also, PCA analysis was employed to achieve a distinguish classification between these three pathogen‐infected samples with the healthy control (Figure 5j). These results indicated the excellent specificity of the sensor array for detecting *F. graminearum*.

## 3 Conclusion

In conclusion, we developed a wearable colorimetric sensor by extrusion printing of a hybrid ink incorporating PDMS‐coated sensing particles within a PDMS matrix. The dye/UiO@PDMS sensing particles were first fabricated to enhance humidity resistance and VOC‐sensing sensitivity. It is achieved through the water‐blocking and VOC‐capture effect of the hydrophobic PDMS coating. Then, after being mixed with the PDMS matrix, the resulting PDMS/DUP hybrid ink exhibited favorable rheological properties and demonstrated satisfactory printability for extrusion printing. The surface PDMS coating helps to disperse particles more effectively within the PDMS matrix, resulting in the production of high particle‐loading films. The printed film, with high particle loading (70 wt%) and low thickness (below 10 µm), adhered well to a flexible PDMS substrate and demonstrated exceptional mechanical stability while maintaining its sensing performance. The printed nine‐element sensor array exhibited improved sensitivity for sub‐ppm VOC detection, as well as impressive resistance to humidity (up to 90% RH). Additionally, it demonstrated the ability to distinguish between eight different VOCs. Furthermore, the wearable sensor arrays were successfully applied in in situ VOC monitoring from wheat leaves, enabling the accurate and early detection of wheat scab (within two days after inoculation). This research opens up new possibilities for developing versatile and efficient wearable VOC sensors with a wide range of applications in various fields.

## 4 Experimental Section

### Synthesis of Dye/UiO@PDMS Composites

The PDMS coating process for dye/UiO was carried out using a straightforward vapor deposition technique. Initially, the PDMS stamp was prepared by mixing PDMS precursor solutions (Sylgard 184A, Dow Corning, USA) and curing agents (Sylgard 184B, Dow Corning, USA) in a 10:1 mass ratio, followed by curing at 70 °C for 30 min. The as‐prepared dye/UiO powder was uniformly spread onto a glass dish, keeping the layer as thin as possible. This dish was then placed in a glass container with a sufficient amount of PDMS stamp. After being sealed and vacuumed, the glass container was positioned in a digital‐temperature‐controlled oven and held at 200 °C for a duration of T minutes. Once naturally cooled to room temperature, the PDMS‐coated samples were obtained.

### Synthesis of PDMS/DUP Hybrid Inks

The PDMS matrix solution was prepared by mixing 182 mg of polydimethylsiloxane (RTV615 A, Momentive, USA) and 18 mg cross‐linking agent (RTV615 B, Momentive, USA) in 2 mL of toluene, resulting in a final concentration of 100 mg mL^−1^. To create the mixed‐matrix ink with varying particle loadings, 40 mg of dye/UiO@PDMS powder was dispersed in 1.0 mL of acetone through sonication for 30 min. Subsequently, the corresponding volume of the PDMS matrix solution was introduced. The combined suspension underwent 60 min of sonication in an ultrasonic bath, and then the acetone was evaporated until the total solution achieved the desired viscosity. The PDMS/DU ink was prepared using the same method.

### Extrusion Printing of Hybrid Inks on the Substrate

The process of extrusion printing was carried out using a programmable three‐axis pneumatic robotic deposition system (SM300ΩX‐3ASS, MUSASHI) controlled by a computer. The software used for designing patterns (MuCAD, MUSASHI) enabled to fine‐tune extrusion printing parameters to achieve various patterns. During the printing procedure, a cylindrical extrusion nozzle could move in 3D at a predetermined speed, following a pre‐programmed printing protocol. The ink was extruded through a narrow cylindrical nozzle (inner diameter of 110 µm, standard size 32 G) at a fixed pressure (5 kPa) and a linear speed of 40 mm s^‒1^. It was deposited directly onto specific substrates such as PDMS, PET, PVDF, and glass under normal environmental conditions. Before printing, polymer and glass substrates underwent a 10‐minute air plasma treatment using a plasma cleaner (PDC‐002, Harrick Plasma) to enhance their wettability. After printing, the printed films were placed in a vacuum drying oven at 60 °C overnight to ensure complete evaporation of the solvent in the ink.

### Fabrication of Flexible Sensor Array

The chemical formula was PDMS/DUP (70 wt%) inks. Employing the same printing method, nine variations of formulated inks, differing from dyes,  were sequentially deposited onto a flexible PDMS substrate, resulting in a batch of sensor arrays. The single sensor array was cut from the batch.

### VOC Sensing

The setup was shown in Figure S21 (Supporting Information). The VOCs were generated by blowing the corresponding liquid using nitrogen (N_2_). The concentration of VOC was regulated by manipulating the gas stream through a mass flow controller (CSC200‐C, Sevenstar, Beijing Sevenstar Electronics Co., Ltd., China) and measured by an FTIR analyzer (MATRIX‐MG5, Bruker, Germany). The sensor arrays were then subjected to a 20‐minute exposure within a VOC flow. The changes of the sensor array before and after exposure were captured using a scanner (V600, EPSON, Japan).

### In Situ Detection of the VOC Markers from Living Wheat Plants

The sensor array was directly attached to the wheat leaves for 30‐minute detection. The images of sensor array before and after exposure were recorded on a scanner (V600, EPSON, Japan). The VOC monitoring of the infected wheat plants and the controls were conducted every 24 h after inoculation over the next several days.

### Data Analysis

Colorimetric detection signals were calculated by subtracting the red, green, and blue (R, G, B) values of each spot in pre‐exposure images from the corresponding values in post‐exposure images. The differential values (ΔR, ΔG, ΔB) of each spot were extracted and computed using Python. To facilitate visual representation, color differential maps were generated by expanding the RGB range from 3−10 to 0−255. For a quantitative description of the detection response, the Euclidean distance (ED) was calculated using the formula ($ED\;=\sqrt{\Delta R^{2}\;+\Delta G^{2}\;+\Delta B^{2}}\;$). The unsupervised statistical method, principal component analysis (PCA), was employed on the test sample database (comprising RGB difference vectors for each spot on the sensor array) using Unscrambler X 10.4 software.

## Conflict of Interest

The authors declare no conflict of interest.

## Supporting information

Supporting Information

## Data Availability

The data that support the findings of this study are available from the corresponding author upon reasonable request.
