# Supplementary material for: Extrusion Printing of Surface‐Functionalized Metal‐Organic Framework Inks for a High‐Performance Wearable Volatile Organic Compound Sensor
Source: Adv Sci (Weinh). 2024 Apr 24;11(25):2400207. doi: 10.1002/advs.202400207 (PMC11220709; doi:10.1002/advs.202400207)
Supplement: Supplementary file 1 — Supporting Information [file ADVS-11-2400207-s001.pdf]

ADVANCED SCIENCE
Open Access

Supporting Information

for *Adv. Sci.*, DOI 10.1002/advs.202400207

Extrusion Printing of Surface-Functionalized Metal-Organic Framework Inks for a High-Performance Wearable Volatile Organic Compound Sensor

*Xiao Wang, Hao Qi, Yuzhou Shao, Mingming Zhao, Huayun Chen, Yun Chen, Yibin Ying and Yixian Wang**

Supporting Information

Extrusion Printing of Surface-Functionalized Metal-Organic Framework Inks for a High-Performance Wearable Volatile Organic Compounds Sensor

Xiao Wang[a,c], *Hao Qi*[d,e], *Yuzhou Shao*[a,c], *Mingming Zhao*[a,c], *Huayun Chen*[a,c], *Yun Chen*[d,e], *Yibin Ying*[a,b,c], *Yixian Wang*[a,b,c,*]

[a] *School of Biosystems Engineering and Food Science, Zhejiang University, Hangzhou 310058, PR China*

[b] *ZJU-Hangzhou Global Scientific and Technological Innovation Center, Hangzhou 310058, PR China*

[c] *Key Laboratory of Intelligent Equipment and Robotics for Agriculture of Zhejiang Province, Hangzhou 310058, PR China*

[d] *State Key Laboratory of Rice Biology, Zhejiang University, Hangzhou 310058, PR China*

[e] *Key Laboratory of Molecular Biology of Crop Pathogens and Insects, Institute of Biotechnology, Zhejiang University, Hangzhou 310058, PR China*

* Corresponding author: Prof. Yixian Wang

E-mail address: yixianwang@zju.edu.cn

Experimental section

Chemicals and materials.

Zirconium Oxychloride Octahydrate ($ZrOCl_2 \cdot 8H_2O$, 98.0%), terephthalic acid (98.0 %), and acetic acid (HAc, 99.0%) were purchased from Sigma-Aldrich. Ethanol (99.9%), toluene (99.5%), and acetone (99.5%) were purchased from Sinopharm Chemical Reagent Co., Ltd. N, N-Dimethylformamide (DMF, 99.0%) was purchased from Alfa Aesar. Bromophenol blue, bromocresol purple, bromothymol blue, bromocresol green, m-cresol purple, acridine orange Base, and cresol red were purchased from Sigma-Aldrich. Bromophenol red and pyrocatechol violet were obtained from TCI. All materials were used as received without further purification.

Synthesis of UiO-66(Zr).

$ZrOCl_2 \cdot 8H_2O$ (1.146 g) and terephthalic acid (0.191 g) were ultrasonically dissolved in 60 mL of DMF. Then, 20 mL of HAc was added and mixed thoroughly. The mixture was sonicated for 10 min and heated at 120 °C for 24 h. After being cooled to room temperature, the powder was collected by centrifugation and washed with DMF and acetone three times. The product was activated by soaking in acetone at 60 °C, changing the acetone every 5 h five times, and then drying at 100 °C under vacuum. Finally, the product was thoroughly dried overnight at 120 °C under vacuum.

Synthesis of dye/UiO.

After dissolving 10 mg of the dye in 50 mL of ethanol, 50 mg of UiO-66(Zr) was introduced into the solution. The mixture was stirred at room temperature for 12 h. The resulting sample was obtained by centrifuging at 9,000 r.p.m for 15 min, followed by three ethanol washes, and finally dried under vacuum at room temperature for 12 h.

Characterization.

Scanning electron microscopy (SEM) images were carried out on a Zeiss G300 scanning electron microscope (Zeiss, Germany). High-resolution transmission electron microscopy (HRTEM) images were observed using a JEOL JEM-2100Plus transmission electron microscope (JEOL, Japan) at an accelerating voltage of 200 kV. Energy-dispersive X-ray spectroscopy (EDS) element mapping images were conducted on an FEI Tecnai G2 F20 S-TWIN transmission electron microscope (FEI, USA) equipped with Oxford X-MAX 80T EDS

detector (Oxford Instruments, UK). Powder X-ray diffractometer (PXRD) patterns were recorded on a Bruker D8 Advance diffractometer (Bruker, Germany) (Cu Kα X-ray radiation, λ = 1.54 Å). Each PXRD pattern was acquired at a voltage of 40 kV and a current of 40 mA. Fourier transform infrared spectroscopy (FT-IR) spectra were measured using a Thermo Scientific NICOLET iS50FT-IR spectrometer (Thermo Fisher Scientific, USA) with a scanning range of 500-3500 cm^{-1} and resolution of 4 cm^{-1}. The static water contact angles were measured using a Biolin Scientific Theta Lite instrument (Biolin Scientific, Sweden). The tensile testing of the printed sensor was performed on a universal testing machine (UTM2102, SUNS, Shenzhen, China).

Testing of the humidity-resistance capacity of the sensor arrays.

The colorimetric sensors were placed in the chamber with different RH for 30 min. The RH of the gas chamber is adjusted by mixing the proper proportion of dry and wet N_2 using digital mass-flow controllers (CSC200-C, Sevenstar, Beijing Sevenstar Electronics Co., Ltd., China) and measured by a digital humidity sensor (Jianda Renke, Shandong Renke Control Technology Co., Ltd., China). The images of the sensor array before and after exposure were recorded using a scanner (V600, EPSON, Japan).

Inoculation of wheat leaves.

Wheat seedlings (Jimai 22) were cultivated in a greenhouse at 25 °C under 12 h of light per day. A typical *Fusarium graminearum* (*F. graminearum*) wild-type strain PH-1 (NRRL 31084) was cultured on potato-dextrose agar (PDA) plate culture media at 25 °C for 4 d. The wheat plant at the stage of 10 d after sowing was inoculated by spraying *F. graminearum* sporangia solution (~50,000 sporangia ml^{-1}) on the whole plant. The inoculated plants were put in an incubator (25 °C, 80 % RH) under 12 h of light per day. The healthy wheat plants were treated as the same operation, used as a control.

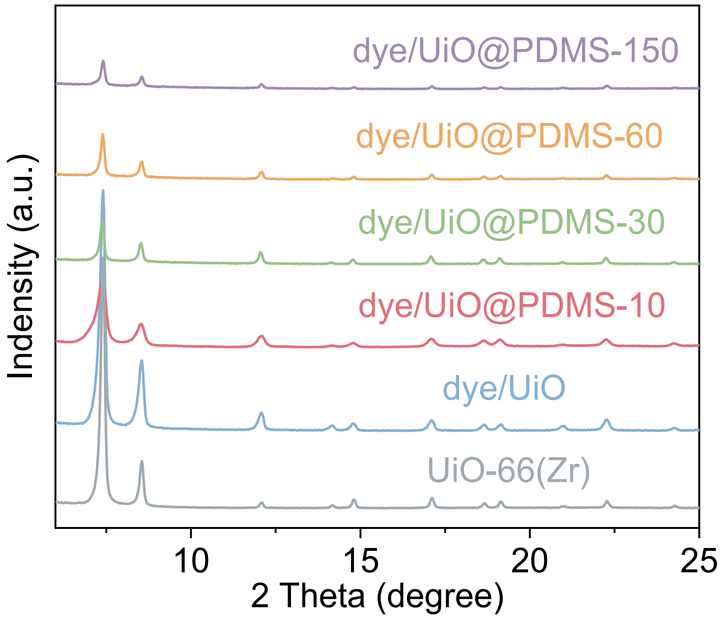

Figure S1. Powder X-ray Diffraction (PXRD) patterns of UiO-66(Zr), dye/UiO, and dye/UiO@PDMS-T with varying coating time of 10, 30, 60, and 150 min.

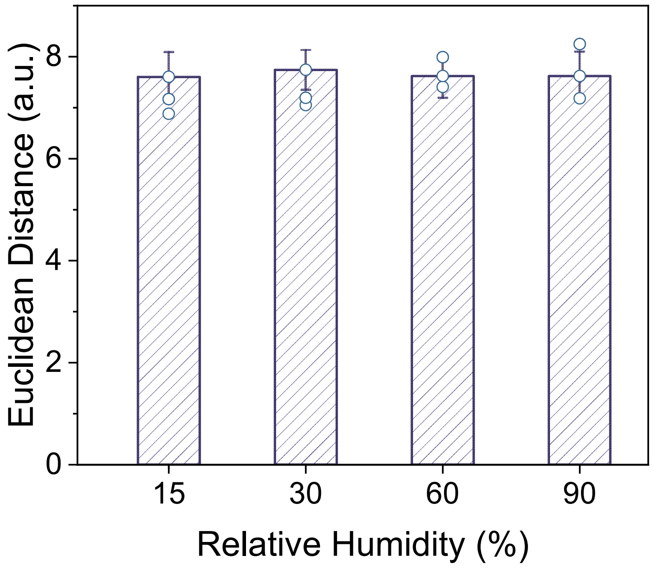

Figure S2. Humidity-independent VOCs sensing performance. The ED values of dye/UiO@PDMS-150 to 10 ppm 1-octence-3-ol at different humidity (15–90% RH).

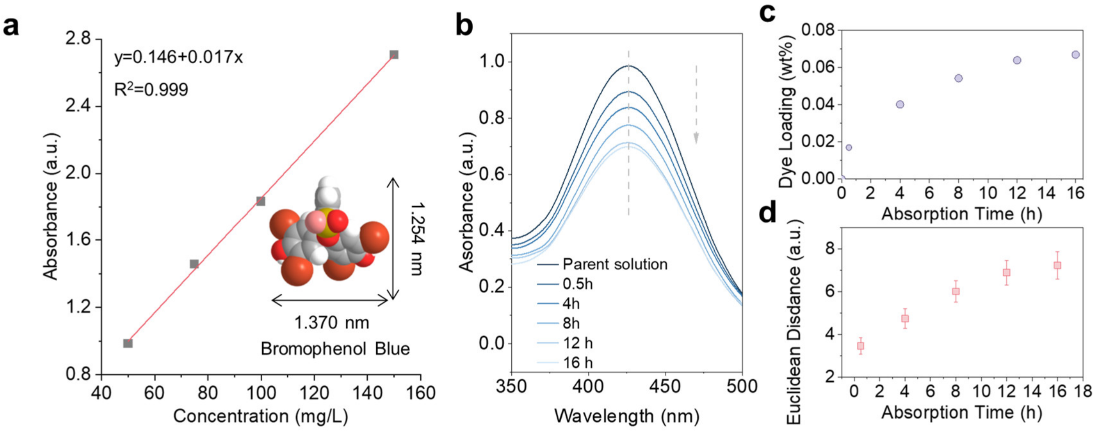

Figure S3. (a) UV-vis calibration curve of bromophenol blue. Inset: structure and molecular size of bromophenol blue. (b) UV-vis spectra of the pristine dye solution and the supernatant after UiO adsorption under different adsorption time. To ensure that the measured values do not exceed the measurement range, all test solutions were diluted to 1/4 of their original concentration. (c) Dye-loading amount after different adsorption time. (d) The ED values of corresponding dye/UiO@PDMS-150 to 10 ppm of 1-octene-3-ol.

The dye uptake was calculated from the following equation:

$$Q_{ad} = \frac{(C_0 - C_{ad})V}{m} \times 100\%\, wt$$

(C_0: initial concentration of dye in ethanol solution (mg/L); C_{ad}: the concentration after adsorption (mg/L); m: the mass of adsorbent MOF (mg); V is the volume of the solution (L)).

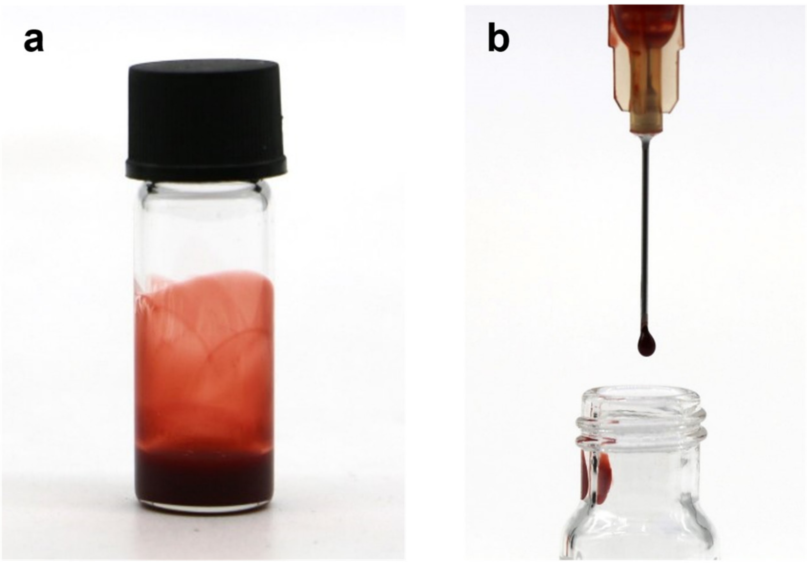

Figure S4. Optical images of (a) PDMS/DUP (70% wt) ink and (b) squeezing ink through a syringe, showing appropriate viscosity and satisfied rheological properties.

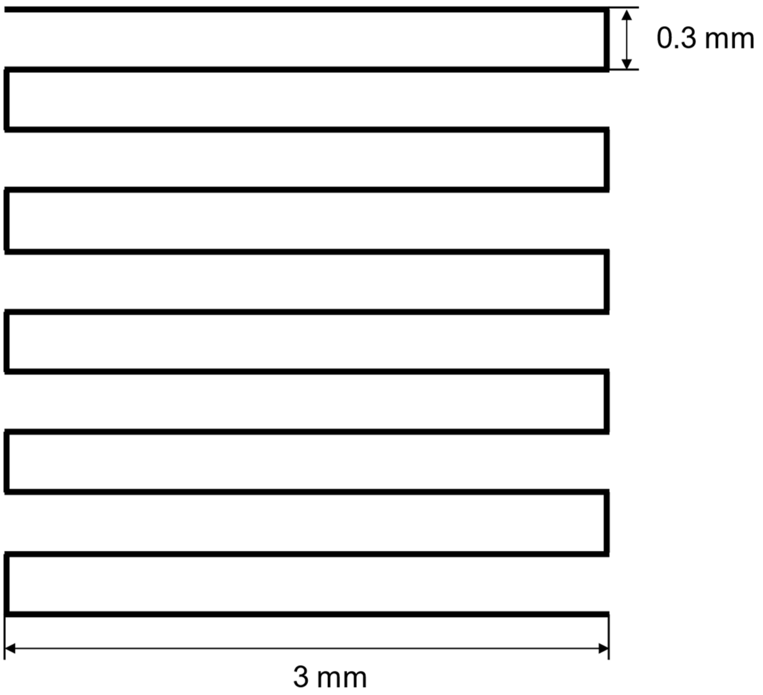

Figure S5. Path of the printing nozzle for preparing the hybrid film (a square with a side length of 3 mm) designed using MuCAD software.

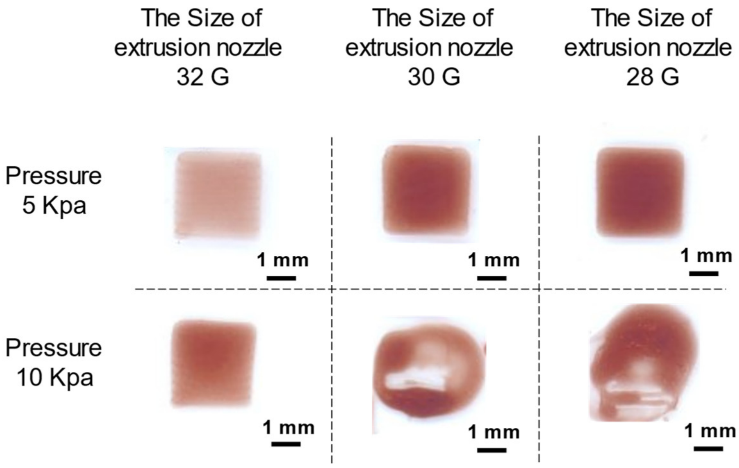

Figure S6. Optical images of PDMS/DUP (70% wt) films (a square with a side length of 3 mm) printed using extrusion nozzles of different sizes and under various N_2 pressures. The corresponding inner diameter for standard sizes of 32 G, 30 G, and 28 G are 100 µm, 150 µm, and 170 µm. Under varying nozzle sizes and extrusion N_2 pressures, the prepared films exhibit different levels of quality. As the nozzle size and N_2 pressure increase, the volume of ink ejected rises, resulting in progressively thicker films. Excessive ink ejection, however, can impede the uniform formation of the film. This phenomenon arises from the higher surface tension of larger ink droplets, which slows down their diffusion rate on the substrate. Consequently, the prolonged evaporation and solidification process complicates the formation of a uniform and stable thin film. To achieve a uniform film, the optimized printing parameters are the nozzle size of 32 G and a pressure of 5 Kpa.

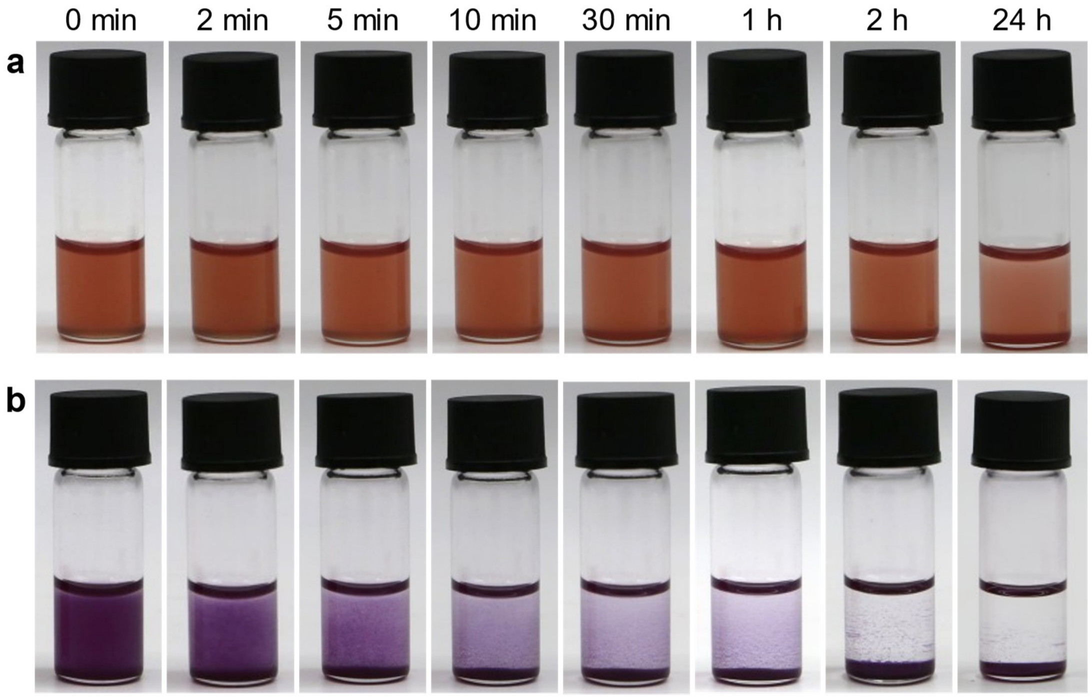

Figure S7. Dispersibility test of (a) dye/UiO@PDMS-150 particles and (b) dye/UiO particles in toluene. ~1 mg particles were dispersed in 1mL toluene after 30-min sonication. Dispersibility was observed at various time after settling the suspension.

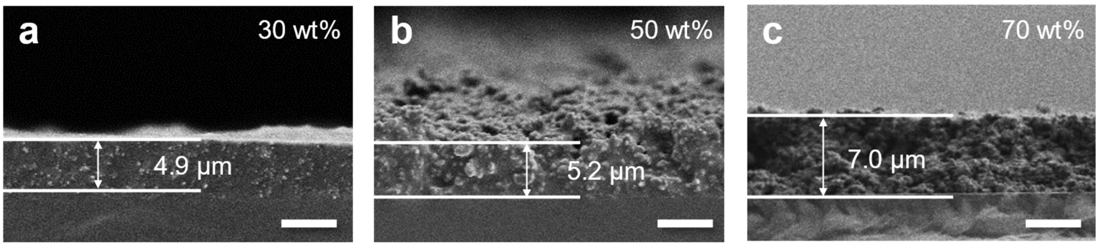

Figure S8. Cross-section SEM images of printed PDMS/DUP film with different dye/UiO@PDMS particle loading (30 wt%, 50 wt%, and 70 wt%). Scale bar: 5 μm.

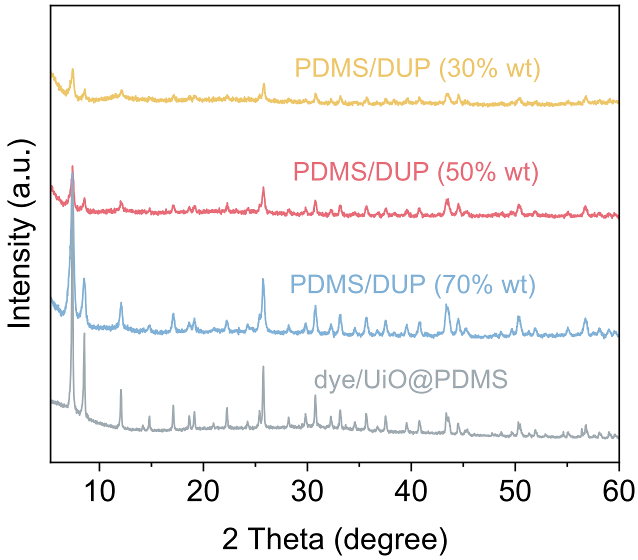

Figure S9. XRD patterns of dye/UiO@PMDS and printed PDMS/DUP films with different dye/UiO@PDMS particle loading (30%wt, 50%wt, and 70%wt).

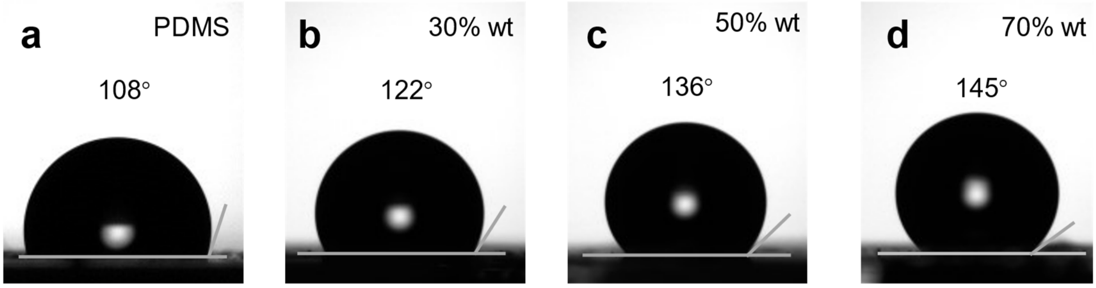

Figure S10. Water contact angle images of PDMS substrate and printed film with different dye/UiO@PDMS particle loading.

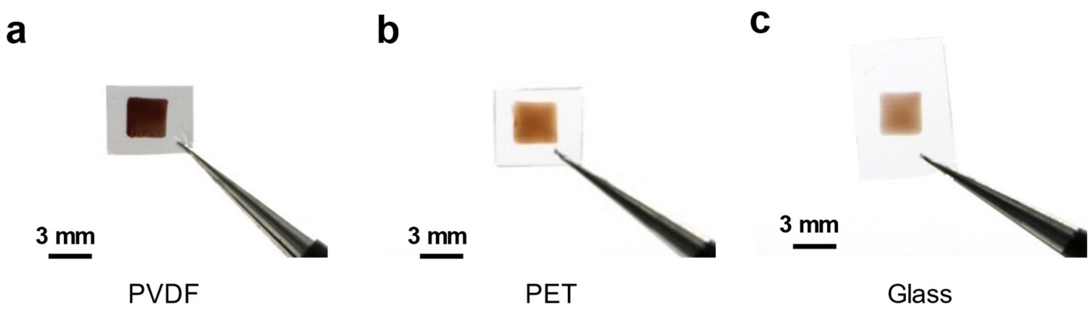

Figure S11. Printability on different substrates. Optical images of printed films (3 mm × 3 mm) on (a) PVDF, (b) PET, and (c) glass.

.

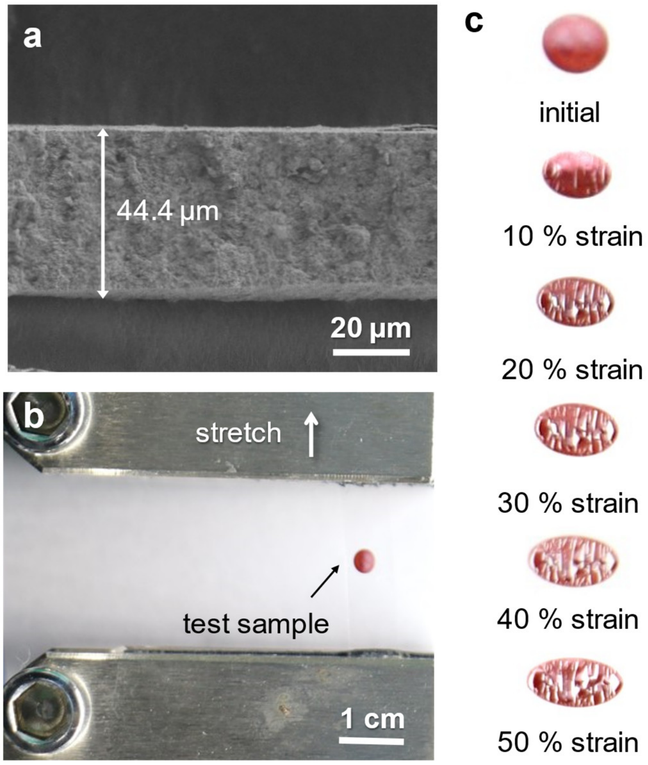

Figure S12. (a) cross-section SEM image of PDMS/DUP (70%wt) film by spin coating. (b) optical image showing the tensile testing. The test sample is prepared by spin-coating of PDMS/DUP (70%wt) ink on a PDMS substrate (the thickness of substrate: 50 μm). (c) Amplifying photographs of the samples in (b) under various strains. The crack appeared when the strain was 10%.

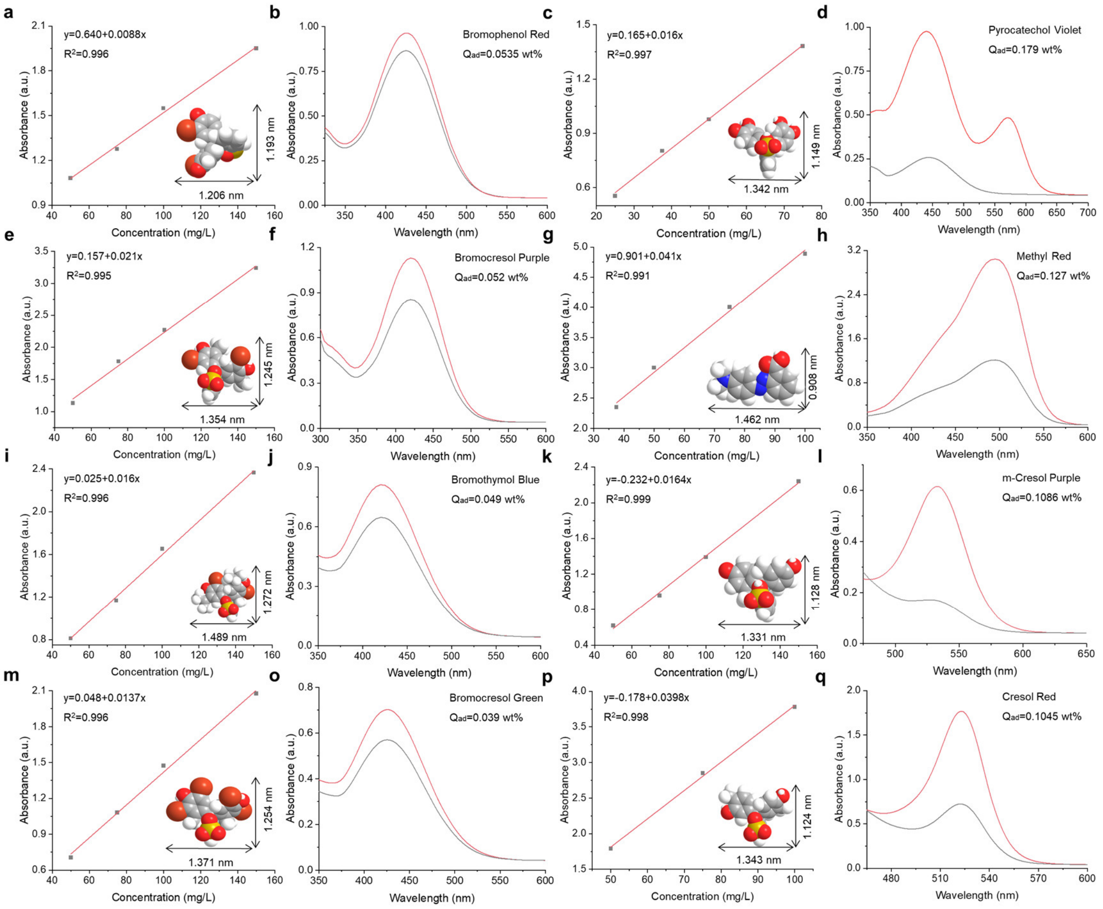

Figure S13. UV-vis calibration curve of dye solution, and UV-vis spectra of dye solutions before (red line) and after (grey line) 12-h exposure to UiO-66 (Zr) at room temperature. The structure and molecular size of corresponding dyes were inserted.

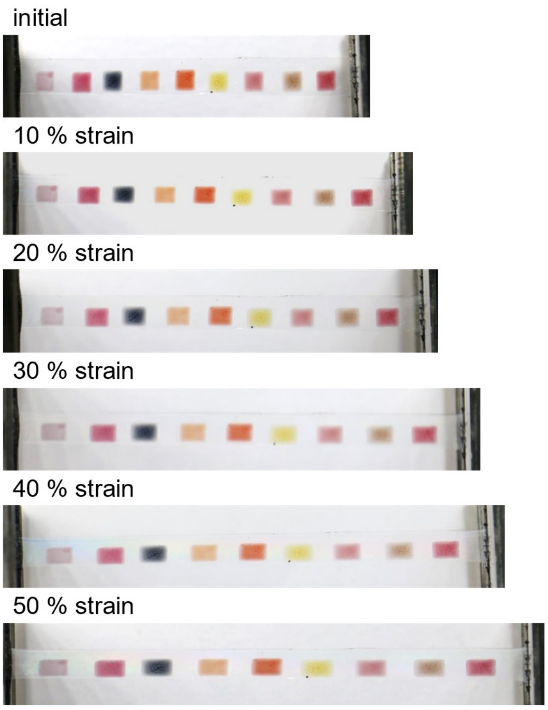

Figure S14. Optical images of the printed sensor under various strains from 0% to 50%.

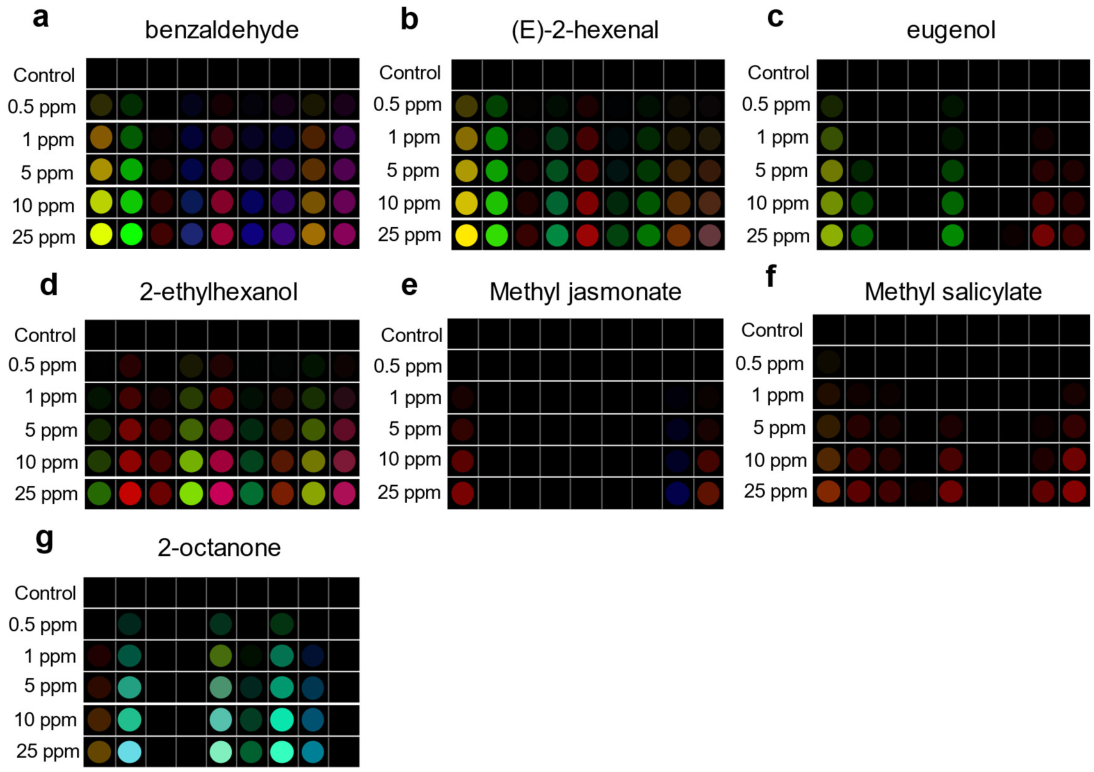

Figure S15. Color differential patterns of the sensor array reflecting the response to (a) benzaldehyde, (b) (E)-2-hexenal, (c) eugenol, (d) 2-ethylhexanol, (e) Methyl jasmonate, (f) Methyl salicylate, and (g) 2-octanone at increasing concentrations from 0.5 to 25 ppm under 20-min exposure. The patterns are obtained from the average of three parallel trials. The RGB color range of 3–10 was expanded to 0–255 for display purposes.

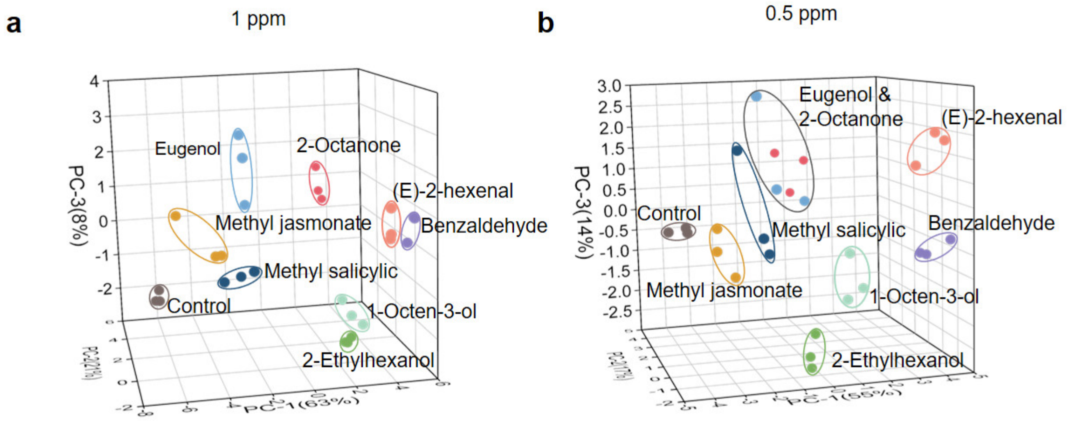

Figure S16. PCA plot with the first three principal componets of 8 plant VOCs and control at (a) 1 and (b) 0.5 ppm, based on three independent experiments of 20-min response to VOCs.

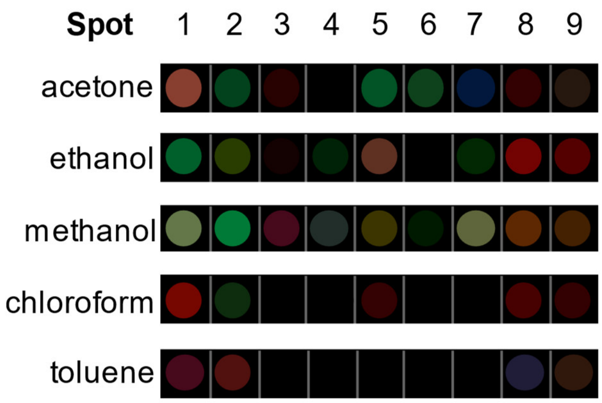

Figure 17. Color differential patterns of the sensor array in response to various generic VOC vapors, including acetone, ethanol, methanol, chloroform, and toluene. The RGB color range of 3–10 was expanded to 0–255 for display purposes.

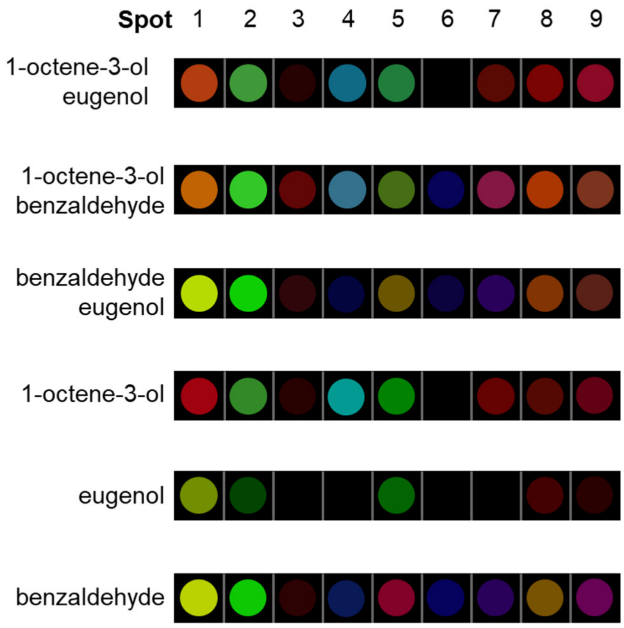

Figure S18. Color differential patterns of the sensor array to VOC mixture and the corresponding single VOC. The RGB color range of 3–10 was expanded to 0–255 for display purposes.

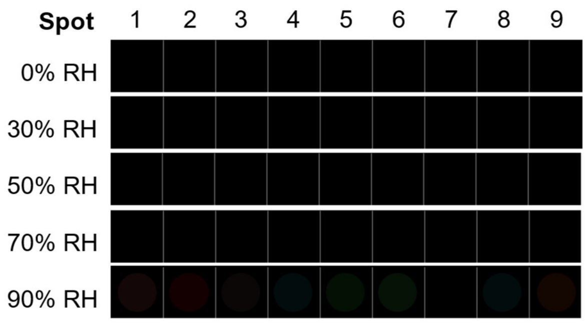

Figure S19. Response of the printed sensor to 30-min water vapor exposure under different RH.

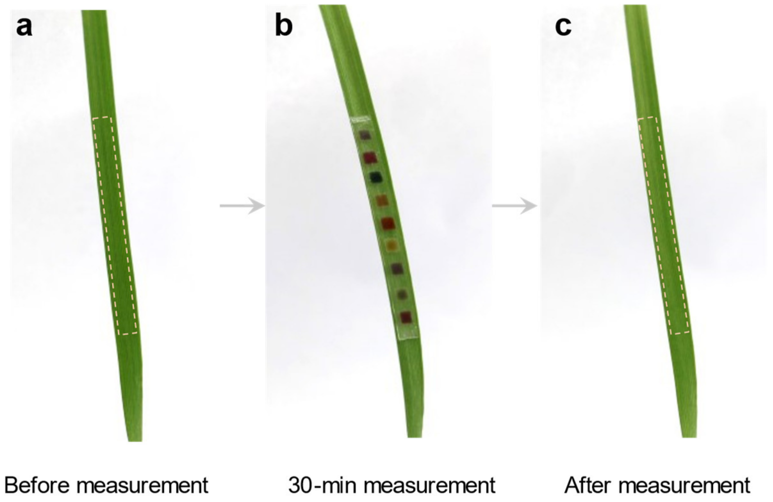

Figure S20. Optical images of the wheat leaf surface before and after applying the sensor. (a) Photo of the wheat leaf before measurement, (b) attached with the sensor, and (c) after measurement. There is no observed physical damage on the wheat leaf after a 30-minute measurement.

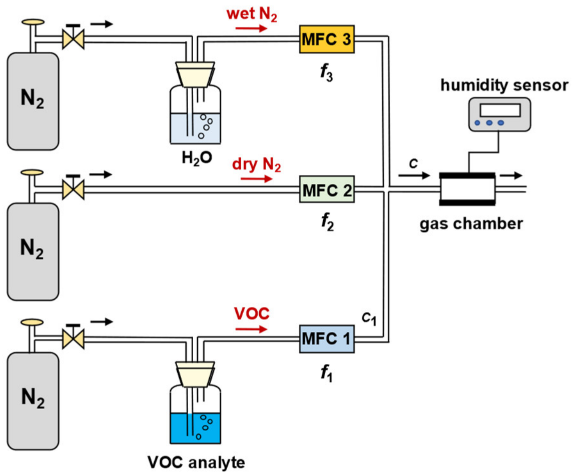

Figure S21. Setup for humidity testing and VOCs detection.

The concentration of diluted VOCs can be controlled using the following equation:

$$\mathrm{c} = \frac{c_1 \times f_1}{f_1 + f_2 + f_3}$$

C: concentration of diluted VOCs (ppm); C_1: concentration of initial VOCs (ppm); f_1: flow rate of MFC_1 (mL/min); f_2: flow rate of MFC_2 (mL/min); f_3: flow rate of MFC_3 (mL/min).

Table S1. The corresponding dye molecules in each spot of sensor arrays.

Spot	Name	Type
1	Bromophenol Blue	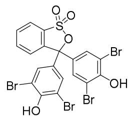
2	Bromophenol Red	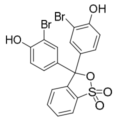
3	Pyrocatechol Violet	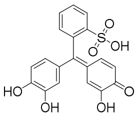
4	Bromocresol Purple	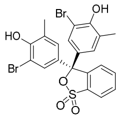
5	Methyl Red	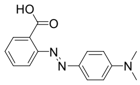
6	Bromothymol Blue	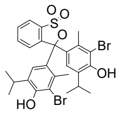
7	m-Cresol Purple	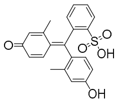
8	Bromocresol Green	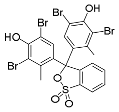
9	Cresol Red	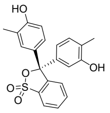

Table S2. Gas sensors utilizing MOF-based thin film.

Sensing Materials	Fabrication Methods	Type	Mechanical Properties	Sensing Performances	Ref.
Pure film					
MOFs	Drop casting	Colorimetric	Rigid	/	S1
Pd/Dye/UiO	Drop casting	Colorimetric	Flexibility, No Stretchability	LOD towards ethylene: ~ 8.2 ppm	S2
Dye/2D MOFs	Drop casting	Colorimetric	Flexibility, No Stretchability	LOR towards 8 VOCs: 15 ppm–5 ppm	S3
dye/MOF@COF	Drop casting	Colorimetric	Flexibility, No Stretchability	LOR towards 8 VOCs: 0.1–1 ppm	S4
$Cu_3(HHTP)_2$	Spay coating	Electrical	Rigid	LOD towards NH_3: 600 ppb	S5
$Cu_3(HHTP)_2$, $Cu_3(HITP)_2$, $Ni_3(HITP)_2$	Drop casting	Electrical	Rigid	The detection range of VOCs: 200–2500 ppm	S6
Hybrid film					
NUS-24 (PEI)	Membrane casting	Fluorescent	Flexibility, No Stretchability	Compared with pure nanosheets, significantly reduced sensing performance in detection of VOC vapors	S7
Tb^{3+}@PI-COF (PVDF)	Membrane casting	Fluorescent	Flexibility, No Stretchability	LOD towards nitrobenzene: 400 ppm	S8
Dye/MOF@PDMS (PDMS)	Extrusion printing	Colorimetric	Flexibility, Stretchability	LOR towards 8 VOCs: 0.5–1 ppm	This work

References

[S1] H. Chen, Z. You, X. Wang, Q. Qiu, Y. Ying, Y. Wang, *Chem. Eng. J.* **2022**, 446, 137098.

[S2] Z. You, M. Zhao, H. Chen, Y. Ying, Y. Wang, *Sens. Actuators, B* **2024**, 399, 134826.

[S3] H. Chen, Z. You, Y. Hong, X. Wang, M. Zhao, Y. Luan, Y. Ying, Y. Wang, *Biosens. Bioelectron.* **2024**, 245, 115826.

[S4] X. Wang, Y. Wang, H. Qi, Y. Chen, W. Guo, H. Yu, H. Chen, Y. Ying, *ACS Nano* **2022**, 16, 14297.

[S5] M. S. Yao, X. J. Lv, Z. H. Fu, W. H. Li, W. H. Deng, G. D. Wu, G. Xu, *Angew. Chem., Int. Ed.* **2017**, 56, 16510.

[S6] M. G. Campbell, S. F. Liu, T. M. Swager, M. Dinca, *J. Am. Chem. Soc.* **2015**, 137, 13780.

[S7] Dong, K. Zhang, X. Li, Y. Qian, H. Zhu, D. Yuan, Q. H. Xu, J. Jiang, D. Zhao, *Nat. Commun.* **2017**, 8, 1142.

[S8] X. Quan, X. Xu, B. Yan, *J. Hazard. Mater.* **2022**, 427, 127869.
